# Immune Checkpoint Inhibitor-Induced Type 1 Diabetes Mellitus in a Patient With Melanoma: A Case Report

**DOI:** 10.7759/cureus.92247

**Published:** 2025-09-13

**Authors:** Mati Shavit

**Affiliations:** 1 Pulmonary Medicine, Soroka Medical Center, Be'er Sheva, ISR; 2 Pulmonary Medicine, Shaare Zedek Medical Center, Jerusalem, ISR

**Keywords:** diabetic ketoacidosis, immune checkpoint inhibitor, immune-related adverse effects, malignant melanoma metastasis, type 1 diabetes mellitus (t1d)

## Abstract

Pembrolizumab, an anti-programmed cell death protein 1 (anti-PD-1) monoclonal antibody, has been approved for use in patients with advanced melanoma and various other indications. Autoimmune diabetes mellitus is one of several immune-related adverse events (irAEs) that have been described with pembrolizumab and other immune checkpoint inhibitor (ICI) therapies. We report the case of a 72-year-old man with no history of diabetes mellitus who was treated with pembrolizumab for advanced melanoma. After six cycles of therapy, he presented to the emergency department with a three-day history of polydipsia, polyuria, and dry mouth. His blood glucose level was 42.9 mmol/L, with a high anion gap metabolic acidosis. A diagnosis of diabetic ketoacidosis (DKA) was made. Treatment with rehydration and intravenous insulin was initiated until recovery, followed by a subcutaneous basal-bolus insulin regimen. Pembrolizumab treatment was continued due to a favorable therapeutic response. ICI-induced type 1 diabetes mellitus is a potentially life-threatening irAE, making blood glucose monitoring imperative during ICI therapy.

## Introduction

The advent of immune checkpoint inhibitors (ICIs) has marked a significant paradigm shift in the treatment of various malignancies, offering durable responses and survival benefits across multiple cancer types [1]. Pembrolizumab, approved by the Food and Drug Administration (FDA) for the treatment of several malignancies, is a monoclonal antibody that binds to programmed cell death protein 1 (PD-1) receptors on T cells and is used for advanced melanoma therapy. PD-1, like other immune checkpoints, is a receptor expressed on immune cells that regulates immune homeostasis. The binding of PD-1 to its ligand, programmed death-ligand 1 (PD-L1), on activated T cells maintains tolerance to self-antigens; as a result, tumor cells exploit this interaction to evade immune surveillance. Thus, while ICIs enhance antitumor immunity, they can also precipitate immune-related adverse events (irAEs), which may affect any organ system. Endocrine irAEs reported with ICIs include thyroid disorders, hypophysitis, primary adrenal insufficiency, and type 1 diabetes mellitus (T1DM). Although T1DM is a rare entity, with an incidence of <1% among patients receiving ICIs, it is a potentially life-threatening irAE if not promptly recognized and managed [2-3]. Here, we report a case of a patient who developed insulin-dependent diabetes mellitus following pembrolizumab treatment for metastatic melanoma.

## Case presentation

**Figure 1:**
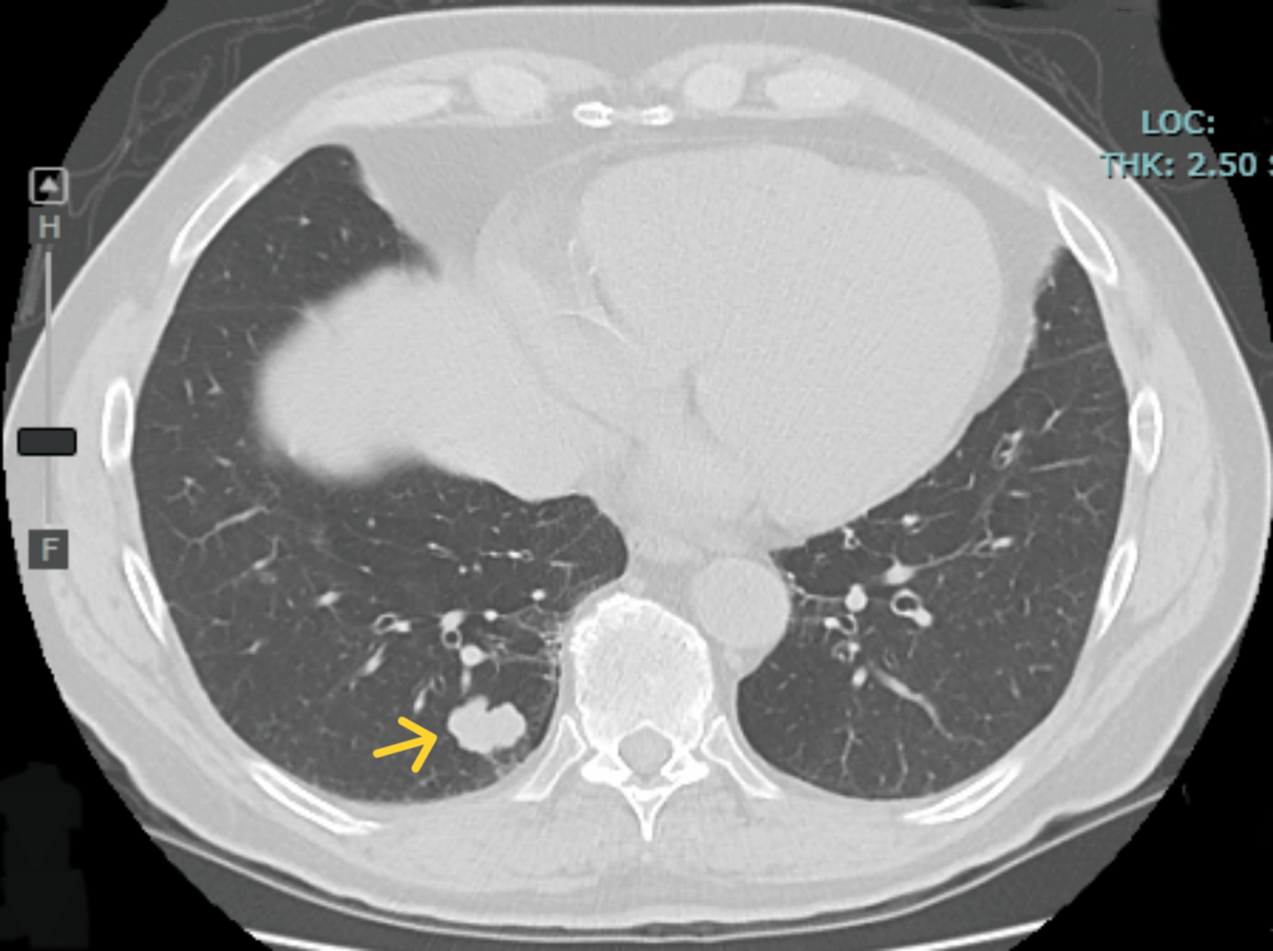
Axial CT of the chest demonstrated a solid pulmonary nodule (yellow arrow) in the right lower lobe CT, computed tomography

A 72-year-old man with a medical history of ischemic heart disease (IHD) and melanoma presented to the emergency department with a three-day history of polydipsia, polyuria, dry mouth, and diarrhea. Eleven years prior to the current presentation, he was diagnosed with nonulcerated melanoma of the left hand, which was surgically excised, and the sentinel lymph node was negative. Ten years later, a solid nodule in the right lung was discovered and surgically removed (Figure 1).

He was diagnosed with pulmonary metastatic malignant melanoma. Pembrolizumab treatment was initiated and administered every three weeks. He received six doses before his current presentation. The family history of endocrine diseases was unremarkable, except for a history of type 2 diabetes mellitus (T2DM) in his mother. On physical examination, he had a lean body mass, was alert and oriented, but clinically dehydrated, tachypneic (26 breaths per minute), and tachycardic (102 beats per minute). He was afebrile, with blood pressure of 133/66 mmHg and oxygen saturation measured 95% on ambient air.

Laboratory analysis revealed hyperglycemia (42.9 mmol/L), pseudohyponatremia (127 mmol/L), moderate hyperkalemia (6.1 mmol/L), and acute renal insufficiency (creatinine clearance 52 mL/min/1.73 m^2^). The presence of urinary ketones, along with a blood gas analysis revealing high anion gap metabolic acidosis, established the diagnosis of DKA. Glutamic acid decarboxylase (GAD) and insulinoma-associated antigen 2 (IA-2) antibodies were within the normal limits (Table 1).

**Table 1:** Laboratory results pCO2, partial pressure of carbon dioxide; CRP, C-reactive protein; HbA1C, glycated hemoglobin; GAD, glutamic acid decarboxylase; IA-2, insulinoma-associated antigen 2; TSH, thyroid-stimulating hormone

Laboratory test	Value	Reference range
Serum glucose (mmol/L)	42.9	4-6
Urine glucose (mg/dL)	>1000	negative-100
Urine ketones (mg/dL)	>80	negative
pH	7.319	7.38-7.42
Bicarbonate (mmol/L)	16.9	22-28
pCO2 (mmHg)	33.5	35-45
Anion gap (mmol/L)	24.9	4-12
Albumin (g/L)	49	32-48
Creatinine (µmol/L)	113	65-115
Potassium (mmol/L)	6.1	3.5-5.1
Sodium (mmol/L)	127	136-145
Lactate (mmol/L)	3.23	0.5-2.2
CRP (mg/dL)	0.23	0-0.5
HbA1c (%)	9	4-5.7
C-peptide (ng/ml)	0.12	0.5-2.5
GAD antibodies (IU/ml)	5.81	<30
IA-2 antibodies	negative	<0.02
TSH (mIU/L)	3.12	0.4-4

Treatment with rehydration and intravenous (IV) insulin was initiated in the emergency department. The patient was admitted to the intermediate care unit in the internal medicine department and was treated with IV insulin and fluids. Serial monitoring of blood glucose and anion gap levels was performed until full recovery, after which a subcutaneous basal-bolus insulin regimen was initiated. Pembrolizumab treatment was continued due to a favorable therapeutic response but was discontinued after six months because of medical insurance constraints.

## Discussion

ICIs include monoclonal antibodies that block cytotoxic T-lymphocyte antigen 4 (CTLA-4) and PD-1 or its ligand, programmed death-ligand 1 (PD-L1), providing remarkable benefits in the treatment of many patients with advanced malignancies. Along with the therapeutic value of ICIs, their increased use has highlighted the importance of awareness of potentially severe irAEs [4]. IrAEs involving the endocrine glands can, in rare cases, cause autoimmune diabetes mellitus, which in most cases presents with DKA [2-3,5].

Our patient exhibited classic signs of DKA, including a three-day history of polydipsia, polyuria, dry mouth, signs of volume depletion, mild hyperventilation on physical examination, and marked hyperglycemia with a high anion gap metabolic acidosis on laboratory tests. However, the development of diabetes was not typical of autoimmune T1DM, as GAD and IA-2 antibodies were not detected. More than 90% of patients with typical T1DM have detectable diabetes-related autoantibodies at diagnosis, compared to approximately half of ICI-treated patients who develop T1DM as an adverse event, as reported in a review of 90 cases of ICI-induced T1DM [6]. In addition, patients with any positive diabetes-related autoantibodies developed diabetes earlier after ICI therapy than those without autoantibodies [3]. Some authors have proposed the two-hit hypothesis, wherein an initial increase in PD-L1 expression in the pancreas likely represents an attempt to suppress an inflammatory response, followed by exposure to ICIs as the second and final trigger for the development of ICI-induced T1DM [7]. These findings highlight our limited understanding of the pathogenic mechanisms underlying ICI-induced T1DM.

It appears that ICI-induced T1DM is more commonly associated with PD-1 or PD-L1 inhibitors than with CTLA-4 inhibitors [5-6,8]. The median time to onset is highly variable, typically ranging from seven to 17 weeks after the first cycle of ICI treatment [8]. However, it typically develops within three months of initial exposure to PD-1 or PD-L1 inhibitors [9]. In this case, ICI-induced T1DM occurred 15 weeks after the first treatment cycle.

Although ICI-induced T1DM is uncommon, it is reasonable to monitor blood glucose levels during ICI therapy to identify potential cases. Suspending treatment may prevent T1DM development or at least preclude the emergence of DKA, as DKA is a frequent presentation of ICI-induced T1DM, which occurs in 40-76% of cases in clinical studies [7]. The American Society of Clinical Oncology Clinical Practice Guideline recommends monitoring patients for hyperglycemia or other signs and symptoms of new or worsening diabetes mellitus, including baseline glucose measurement, testing at each treatment cycle for the first 12 weeks, and then every three to six weeks thereafter [10]. The management of ICI-induced T1DM is not well characterized. While other irAEs are often treated with corticosteroid immunosuppression, this approach is not recommended for ICI-induced T1DM, as corticosteroids can worsen blood glucose levels. Experts recommend suspending ICI therapy in patients who present with DKA [10-11] and rechallenging treatment once glycemic control with insulin therapy is achieved. In most reported cases, including this one, restarting immunotherapy did not worsen glycemic control [12].

## Conclusions

ICIs have become essential in the treatment of various malignancies; however, their increased use has been associated with significant irAEs. ICI-induced T1DM is a rare irAE that can be life-threatening and often presents initially as diabetic ketoacidosis, as observed in this case. Given the growing number of patients receiving ICI therapy and the accessibility of blood glucose monitoring, clinicians should remain vigilant, and patients should be educated about this rare but serious complication.
